# Supplementary figures and images for: Sex disparities in the associations of overall versus abdominal obesity with the 10-year cardiovascular disease risk: Evidence from the Indonesian National Health Survey
Source: PLoS One. 2024 Sep 23;19(9):e0307944. doi: 10.1371/journal.pone.0307944 (PMC11419361; doi:10.1371/journal.pone.0307944)

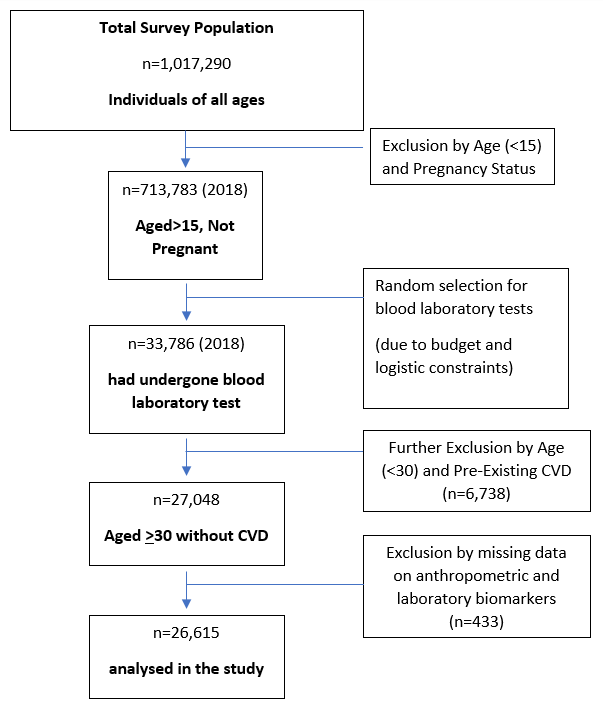


**Supplemental Figure 1. Study Flow Chart**

Supplement: S1 Fig — (DOCX) [file pone.0307944.s001.docx]
