# Supplementary material for: Sex disparities in the associations of overall versus abdominal obesity with the 10-year cardiovascular disease risk: Evidence from the Indonesian National Health Survey
Source: PLoS One. 2024 Sep 23;19(9):e0307944. doi: 10.1371/journal.pone.0307944 (PMC11419361; doi:10.1371/journal.pone.0307944)
Supplement: S1 Table — a and b. Descriptive Characteristics of the Indonesian Population, as Stratified by Urban/Rural Living Situation and Obesity Phenotypes (n = 26,615). (DOCX) [file pone.0307944.s002.docx]

**Supplemental Table 1a. Descriptive Characteristics of the Indonesian Population, Urban/Rural-Stratified (n=26,615)**

|  | **Total (100%)** | **Urban (54.2%)** | **Rural (45.8%)** |
| --- | --- | --- | --- |
| Age, *years* | 49.3 (12.3) | 48.7 (11.5) | 49.9 (13.1) |
| Sex, *%Men* | 54.2 | 50.1 | 51.1 |
| Education, *%High* | 5.7 | 8.6 | 2.5 |
| Occupation, *%Unemployed* | 30.2 | 35.7 | 23.7 |
| Marital Status, *%Married* | 85.4 | 84.7 | 86.1 |
| Smoking, *%yes* | 35.2 | 32.2 | 38.6 |
|  |  |  |  |
| Systolic Blood Pressure, *mmHg* | 135.8 (24.7) | 135.5 (23.8) | 136.2 (25.7) |
| Diastolic Blood Pressure, *mmHg* | 85.9 (13.2) | 86.4 (12.8) | 85.2 (13.5) |
| HDL Cholesterol, *mg/dL* | 47.6 (11.4) | 47.5 (11.0) | 47.7 (11.7) |
| LDL Cholesterol, *mg/dL* | 125.9 (33.4) | 127.8 (32.5) | 123.6 (34.2) |
| Total Cholesterol, *mg/dL* | 186.4 (39.1) | 188.6 (37.7) | 183.7 (40.4) |
| Triglyceride, *mg/dL* | 137.5 (100.0) | 141.7 (102.5) | 132.5 (95.2) |
|  |  |  |  |
| BMI, *kg/m^2^* | 24.1 (4.7) | 24.8 (4.6) | 23.3 (4.6) |
| Overall Obesity, *%yes* | 38.1 | 44.4 | 30.6 |
| Waist Circumference, *cm* | 81.4 (12.1) | 83.6 (11.5) | 78.7 (12.2) |
| Abdominal Obesity, *%yes* | 39.4 | 46.5 | 30.9 |

Data were presented as mean (SD) for continuous variables, or proportion (%) for categorical variables.

**Supplemental Table 1b. Descriptive Characteristics of the Indonesian Population, Stratified by Obesity Phenotypes (n=26,615)**

|  | **OO-, AO-**  (53.2%) | **OO+, AO-**  (7.4%) | **OO-, AO+**  (8.7%) | **OO+, AO+**  (30.7%) |
| --- | --- | --- | --- | --- |
| Age, *years* | 50.3 (12.7) | 46.2 (10.1) | 51.9 (13.4) | 47.4 (10.9) |
| Sex, *%Men* | 66.9 | 62.8 | 19.5 | 28.3 |
| Education, *%High* | 4.3 | 7.9 | 5.9 | 7.8 |
| Occupation, *%Unemployed* | 22.3 | 22.8 | 45.4 | 41.5 |
| Marital Status, *%Married* | 85.3 | 89.7 | 77.0 | 86.8 |
| Smoking, *%yes* | 49.1 | 37.5 | 13.5 | 16.6 |
|  |  |  |  |  |
| Systolic Blood Pressure, *mmHg* | 132.1 (23.0) | 135.7 (22.0) | 140.0 (28.0) | 141.1 (26.2) |
| Diastolic Blood Pressure, *mmHg* | 82.7 (12.0) | 87.0 (11.9) | 87.1 (13.7) | 90.8 (13.7) |
| HDL Cholesterol, *mg/dL* | 48.4 (11.4) | 45.2 (10.5) | 49.1 (12.1) | 46.4 (11.1) |
| LDL Cholesterol, *mg/dL* | 120.4 (30.9) | 127.3 (31.8) | 130.7 (37.6) | 133.8 (35.1) |
| Total Cholesterol, *mg/dL* | 179.8 (36.4) | 186.5 (36.5) | 194.4 (43.5) | 195.3 (40.8) |
| Triglyceride, *mg/dL* | 122.9 (80.4) | 154.1 (105.5) | 146.6 (116.5) | 156.0 (120.8) |
|  |  |  |  |  |
| BMI, *kg/m^2^* | 20.9 (2.3) | 26.9 (2.7) | 23.2 (1.6) | 29.3 (3.8) |
| Overall Obesity, *%yes* | 0 | 100 | 0 | 100 |
| Waist Circumference, *cm* | 73.4 (7.1) | 80.0 (7.0) | 87.1 (8.7) | 93.9 (9.3) |
| Abdominal Obesity, *%yes* | 0 | 0 | 100 | 100 |

**OO, Overall Obesity; AO, Abdominal Obesity.** Data were presented as mean (SD) for continuous variables, or proportion (%) for categorical variables.
