# Supplementary material for: Sex disparities in the associations of overall versus abdominal obesity with the 10-year cardiovascular disease risk: Evidence from the Indonesian National Health Survey
Source: PLoS One. 2024 Sep 23;19(9):e0307944. doi: 10.1371/journal.pone.0307944 (PMC11419361; doi:10.1371/journal.pone.0307944)
Supplement: S2 Table — a and b. The Associations of Overall and Abdominal Obesity with the 10-Year Cardiovascular Diseases Risks, as Stratified by Urban/Rural Living Situation and Age Group (n = 26,615). (DOCX) [file pone.0307944.s003.docx]

**Supplemental Table 2a. The Associations of Overall and Abdominal Obesity with the Urban/Rural-stratified 10-Year Cardiovascular Diseases Risks (n=26,615)**

|  |  | **Urban (54.2%)** | **Rural (45.8%)** |
| --- | --- | --- | --- |
| **Overall Obesity** | Crude β (95% CI) | -0.26 (-0.44,-0.08) | -1.34 (-1.52,-1.14) |
| (BMI per SD) | Adjusted β (95% CI) | 1.29 (1.15-1.43) | 1.54 (1.40-1.68) |
| SD = 4.7 kg/m^2^ | +Waist Circumference | 0.56 (0.35-0.78) | 0.81 (0.61-1.02) |
|  |  |  |  |
|  |  | **Urban (54.2%)** | **Rural (45.8%)** |
| **Abdominal Obesity** | Crude β (95% CI) | 1.25 (1.05-1.46) | 0.22 (0.01-0.42) |
| (Waist Circumference per SD) | Adjusted β (95% CI) | 1.38 (1.25-1.51) | 1.45 (1.32-1.59) |
| SD = 12.1 cm | +BMI | 0.94 (0.72-1.16) | 0.90 (0.71-1.10) |

Data were presented as regression coefficient (β) with 95% confidence interval (CI). Multivariate models were adjusted for age, sex, education, occupation, marital status, and living situation (urban/rural). The associations were additionally mutually adjusted for waist circumference and BMI.

**Supplemental Table 2b. The Associations of Overall and Abdominal Obesity with the Age-stratified 10-Year Cardiovascular Diseases Risks (n=26,615)**

|  |  | **Age Group (Years)** | | | | | |
| --- | --- | --- | --- | --- | --- | --- | --- |
|  |  | **30-39** | **40-49** | **50-59** | **60-69** | **70-79** | ***>*80** |
|  |  | **(25.2%)** | **(29.4%)** | **(24.9%)** | **(14.1%)** | **(5.2%)** | **(1.2%)** |
| **Overall Obesity** | Crude β (95% CI) | 0.31 (0.22-0.40) | 0.64 (0.49-0.80) | 0.88 (0.65-1.11) | 0.66 (0.35-0.96) | 0.54 (0.01-1.06) | 0.09  (-1.07,  1.25) |
| (BMI per SD) | Adjusted β (95% CI) | 0.64 (0.53-0.75) | 1.43 (1.27-1.59) | 1.97 (1.73-2.22) | 1.52 (1.24-1.81) | 1.81 (1.34-2.28) | 0.90  (-0.10,  1.91) |
| SD = 4.7 kg/m^2^ | +Waist Circumference | 0.41 (0.25-0.56) | 0.69 (0.45-0.93**)** | 0.84 (0.47-1.21) | 0.60 (0.17-1.02) | 1.30 (0.68-1.92) | 1.08  (-0.18,  2.33) |
|  |  |  |  |  |  |  |  |
|  |  | **30-39** | **40-49** | **50-59** | **60-69** | **70-79** | ***>*80** |
| **Abdominal Obesity** | Crude β (95% CI) | 0.54 (0.45-0.64) | 1.25 (1.09-1.42) | 1.61 (1.39-1.84) | 1.25 (0.95-1.54) | 0.84 (0.48-1.20) | 0.21  (-0.65  ,1.07) |
| (Waist Circumference per SD) | Adjusted β (95% CI) | 0.64 (0.55-0.73) | 1.45 (1.29-1.61) | 2.00 (1.79-2.22) | 1.57 (1.30-1.84) | 1.24 (0.90-1.59) | 0.46  (-0.36  ,1.28) |
| SD = 12.1 cm | +BMI | 0.31 (0.17-0.45) | 0.95 (0.70-1.20) | 1.40 (1.07-1.73) | 1.16 (0.76-1.57) | 0.58 (0.15-1.01) | -0.19  (-1.22  ,0.84) |

Data were presented as regression coefficient (β) with 95% confidence interval (CI). Multivariate models were adjusted for age, sex, education, occupation, marital status, and living situation (urban/rural). The associations were additionally mutually adjusted for waist circumference and BMI.
