# Supplementary material for: Sex disparities in the associations of overall versus abdominal obesity with the 10-year cardiovascular disease risk: Evidence from the Indonesian National Health Survey
Source: PLoS One. 2024 Sep 23;19(9):e0307944. doi: 10.1371/journal.pone.0307944 (PMC11419361; doi:10.1371/journal.pone.0307944)
Supplement: Attachment — Submitted filename: PONE-D-24-16324_ob.edits.pdf [file pone.0307944.s004.pdf]

PLOS ONE

Sex Disparities in the Associations of Overall versus Abdominal Obesity with the 10-Year Cardiovascular Disease Risk: Evidence from the Indonesian National Health Survey

--Manuscript Draft--

**Manuscript Number:**	PONE-D-24-16324
**Article Type:**	Research Article
**Full Title:**	Sex Disparities in the Associations of Overall versus Abdominal Obesity with the 10-Year Cardiovascular Disease Risk: Evidence from the Indonesian National Health Survey
**Short Title:**	Sex Disparities in the Associations of Overall versus Abdominal Obesity with the 10-Year CVD Risk
**Corresponding Author:**	Fathimah Sulistyowati Sigit, MD, PhD<br>Universitas Indonesia Faculty of Public Health: Universitas Indonesia Fakultas Kesehatan Masyarakat<br>Depok, Jawa Barat INDONESIA
**Keywords:**	abdominal obesity; BMI; waist circumference; cardiovascular diseases; visceral fat
**Abstract:**	Background<br>Cardiovascular diseases (CVDs) are a leading cause of disability-adjusted life years in Indonesia. Although obesity is a known risk factor for CVDs, the relative contributions of overall versus abdominal obesity are less clear. We aimed to estimate the 10-year CVD risks of the Indonesian population and investigate the separate and joint associations of overall and abdominal obesity with these risks.<br><br>Methods<br>Using nationally representative data from the Indonesian Health Survey (n=33,786), the 10-year CVD risk was estimated using the Framingham Score. The score was calculated as %-risk, with >20% indicating high risk. Overall obesity was measured by BMI, while abdominal obesity was measured by waist circumference. We performed sex-stratified multivariable linear regressions to examine the associations of standardized units of BMI and waist circumference with the 10-year CVD risk, mutually adjusted for waist circumference and BMI.<br><br>Results<br>Mean (SD) 10-year CVD risks were 14.3(8.9)% in men and 8.0(9.3)% in women, with 37.3% of men and 14.1% of women having high (>20%) risks. After mutual adjustment, one SD in BMI and waist circumference were associated with 0.75(0.50-1.01) and 0.95(0.72-1.18) increase in the %-risk of CVD in men, whereas in women, the β(95% CIs) were 0.43(0.25-0.61) and 1.06(0.87-1.26).<br><br>Conclusion<br>Abdominal fat accumulation showed stronger associations with 10-year CVD risks than overall adiposity, particularly in women. Although men had higher overall CVD risks, women experienced more detrimental cardiovascular effects of obesity. Raising awareness of abdominal/visceral obesity and its more damaging cardiovascular effects in women is crucial in preventing CVD-related morbidity and mortality.
**Order of Authors:**	Fathimah Sulistyowati Sigit, MD, PhD
	Dicky Levenus Tahapary, MD, PhD
	Woro Riyadina, PhD
	Kusharisupeni Djokosujono, Professor
**Additional Information:**	
**Question**	**Response**
**Financial Disclosure**	Yes

Enter a financial disclosure statement that describes the sources of funding for the work included in this submission. Review the submission guidelines for detailed requirements. View published research articles from *PLOS ONE* for specific examples.<br><br>This statement is required for submission and **will appear in the published article** if the submission is accepted. Please make sure it is accurate.<br><br>**Funded studies**<br>Enter a statement with the following details:<br>• Initials of the authors who received each award<br>• Grant numbers awarded to each author<br>• The full name of each funder<br>• URL of each funder website<br>• Did the sponsors or funders play any role in the study design, data collection and analysis, decision to publish, or preparation of the manuscript?<br><br>Did you receive funding for this work?	
Please add funding details.<br>as follow-up to "**Financial Disclosure**<br><br>Enter a financial disclosure statement that describes the sources of funding for the work included in this submission. Review the submission guidelines for detailed requirements. View published research articles from *PLOS ONE* for specific examples.<br><br>This statement is required for submission and **will appear in the published article** if the submission is accepted. Please make sure it is accurate.	The publication of this paper is funded by the Directorate of Research and Development, Universitas Indonesia, under Hibah PUTI 2024.

Powered by Editorial Manager® and ProduXion Manager® from Aries Systems Corporation

**Funded studies**<br>Enter a statement with the following details:<br>• Initials of the authors who received each award<br>• Grant numbers awarded to each author<br>• The full name of each funder<br>• URL of each funder website<br>• Did the sponsors or funders play any role in the study design, data collection and analysis, decision to publish, or preparation of the manuscript?<br><br>Did you receive funding for this work?"	
Please select the country of your main research funder (please select carefully as in some cases this is used in fee calculation).<br>as follow-up to "**Financial Disclosure**<br><br>Enter a financial disclosure statement that describes the sources of funding for the work included in this submission. Review the submission guidelines for detailed requirements. View published research articles from *PLOS ONE* for specific examples.<br><br>This statement is required for submission and **will appear in the published article** if the submission is accepted. Please make sure it is accurate.<br><br>**Funded studies**<br>Enter a statement with the following details:<br>• Initials of the authors who received each award<br>• Grant numbers awarded to each author<br>• The full name of each funder<br>• URL of each funder website<br>• Did the sponsors or funders play any role in the study design, data collection and analysis, decision to publish, or preparation of the manuscript?<br><br>Did you receive funding for this work?"	INDONESIA - ID
**Competing Interests**	The authors have declared that no competing interests exist.

Powered by Editorial Manager® and ProduXion Manager® from Aries Systems Corporation

Use the instructions below to enter a competing interest statement for this submission. On behalf of all authors, disclose any competing interests that could be perceived to bias this work—acknowledging all financial support and any other relevant financial or non-financial competing interests.<br><br>This statement is required for submission and **will appear in the published article** if the submission is accepted. Please make sure it is accurate and that any funding sources listed in your Funding Information later in the submission form are also declared in your Financial Disclosure statement.<br><br>View published research articles from *PLOS ONE* for specific examples.<br><br>**NO authors have competing interests**<br><br>Enter: *The authors have declared that no competing interests exist.*<br><br>**Authors with competing interests**<br><br>Enter competing interest details beginning with this statement:<br><br>*I have read the journal's policy and the authors of this manuscript have the following competing interests: [insert competing interests here]*<br><br>* typeset	
**Ethics Statement**<br><br>Enter an ethics statement for this submission. This statement is required if the study involved:<br><br>• Human participants<br>• Human specimens or tissue<br>• Vertebrate animals or cephalopods<br>• Vertebrate embryos or tissues	All participants signed written informed consent. The ethics of this study have been reviewed and approved by The<br>Research and Community Engagement Ethical Committee, Faculty of<br>Public Health, Universitas Indonesia (No: Ket- 152/UN2.F10.D11/PPM.00.02/2024).

Powered by Editorial Manager® and ProduXion Manager® from Aries Systems Corporation

• Field research<br><br>Write "N/A" if the submission does not require an ethics statement.<br><br>General guidance is provided below. Consult the submission guidelines for detailed instructions. **Make sure that all information entered here is included in the Methods section of the manuscript.**	

Powered by Editorial Manager® and ProduXion Manager® from Aries Systems Corporation

**Format for specific study types**<br><br>**Human Subject Research (involving human participants and/or tissue)**<br>• Give the name of the institutional review board or ethics committee that approved the study<br>• Include the approval number and/or a statement indicating approval of this research<br>• Indicate the form of consent obtained (written/oral) or the reason that consent was not obtained (e.g. the data were analyzed anonymously)<br><br>**Animal Research (involving vertebrate animals, embryos or tissues)**<br>• Provide the name of the Institutional Animal Care and Use Committee (IACUC) or other relevant ethics board that reviewed the study protocol, and indicate whether they approved this research or granted a formal waiver of ethical approval<br>• Include an approval number if one was obtained<br>• If the study involved *non-human primates*, add *additional details* about animal welfare and steps taken to ameliorate suffering<br>• If anesthesia, euthanasia, or any kind of animal sacrifice is part of the study, include briefly which substances and/or methods were applied<br><br>**Field Research**<br><br>Include the following details if this study involves the collection of plant, animal, or other materials from a natural setting:<br>• Field permit number<br>• Name of the institution or relevant body that granted permission	
**Data Availability**<br><br>Authors are required to make all data underlying the findings described fully available, without restriction, and from the time of publication. PLOS allows rare exceptions to address legal and ethical concerns. See the PLOS Data Policy and FAQ for detailed information.	No - some restrictions will apply

A Data Availability Statement describing where the data can be found is required at submission. Your answers to this question constitute the Data Availability Statement and **will be published in the article**, if accepted.<br><br>**Important:** Stating 'data available on request from the author' is not sufficient. If your data are only available upon request, select 'No' for the first question and explain your exceptional situation in the text box.<br><br>Do the authors confirm that all data underlying the findings described in their manuscript are fully available without restriction?	
**Describe where the data may be found in full sentences. If you are copying our sample text, replace any instances of XXX with the appropriate details.**<br><br>• If the data are **held or will be held in a public repository**, include URLs, accession numbers or DOIs. If this information will only be available after acceptance, indicate this by ticking the box below. For example: *All XXX files are available from the XXX database (accession number(s) XXX, XXX.).*<br>• If the data are all contained **within the manuscript and/or Supporting Information files**, enter the following: *All relevant data are within the manuscript and its Supporting Information files.*<br>• If neither of these applies but you are able to provide **details of access elsewhere**, with or without limitations, please do so. For example:<br><br>*Data cannot be shared publicly because of [XXX]. Data are available from the XXX Institutional Data Access / Ethics Committee (contact via XXX) for researchers who meet the criteria for access to confidential data.*<br><br>*The data underlying the results presented in the study are available from (include the name of the third party*	The dataset analysed in this study is available on reasonable request from the Ministry of Health, Republic of Indonesia, subject to prior review.

Powered by Editorial Manager® and ProduXion Manager® from Aries Systems Corporation

and contact information or URL).<br>• This text is appropriate if the data are owned by a third party and authors do not have permission to share the data.<br><br>* typeset	
Additional data availability information:	

Powered by Editorial Manager® and ProduXion Manager® from Aries Systems Corporation

Manuscript
Click here to access/download;Manuscript;Final Manuscript.docx

Sex Disparities in the Associations of Overall versus Abdominal Obesity with the 10-Year Cardiovascular Disease Risk: Evidence from the Indonesian National Health Survey

Fathimah S Sigit[1,2*], Dicky L Tahapary[2,3], Woro Riyadina[4], Kusharisupeni Djokosudjono[1]

[1]Department of Public Health Nutrition, Faculty of Public Health - Universitas Indonesia, Depok, Indonesia

[2]Metabolic Disorder, Cardiovascular, and Aging Cluster, Indonesia Medical Education and Research Institute, Faculty of Medicine - Universitas Indonesia, Jakarta, Indonesia

[3]Department of Internal Medicine, Dr. Cipto Mangunkusumo National Referral Hospital, Faculty of Medicine - Universitas Indonesia, Jakarta, Indonesia

[4]National Research and Innovation Agency, Jakarta, Indonesia

**Corresponding Author*. Correspond to: fathimah10@ui.ac.id.

Main Text Word Count: 3,270

Background

Cardiovascular diseases (CVDs) are a leading cause of disability-adjusted life years in Indonesia. Although obesity is a known risk factor for CVDs, the relative contributions of overall versus abdominal obesity are less clear. We aimed to estimate the 10-year CVD risks of the Indonesian population and investigate the separate and joint associations of overall and abdominal obesity with these risks.

Methods

Using nationally representative data from the Indonesian Health Survey (n=33,786), the 10-year CVD risk was estimated using the Framingham Score. The score was calculated as %-risk, with >20% indicating high risk. Overall obesity was measured by BMI, while abdominal obesity was measured by waist circumference. We performed sex-stratified multivariable linear regressions to examine the associations of standardized units of BMI and waist circumference with the 10-year CVD risk, mutually adjusted for waist circumference and BMI.

Results

Mean (SD) 10-year CVD risks were 14.3(8.9)% in men and 8.0(9.3)% in women, with 37.3% of men and 14.1% of women having high (>20%) risks. After mutual adjustment, one SD in BMI and waist circumference were associated with 0.75(0.50-1.01) and 0.95(0.72-1.18) increase in the %-risk of CVD in men, whereas in women, the β(95% CIs) were 0.43(0.25-0.61) and 1.06(0.87-1.26).

Conclusion

Abdominal fat accumulation showed stronger associations with 10-year CVD risks than overall adiposity, particularly in women. Although men had higher overall CVD risks, women experienced more detrimental cardiovascular effects of obesity. Raising awareness of abdominal/visceral obesity and its more damaging cardiovascular effects in women is crucial in preventing CVD-related morbidity and mortality.

Keywords: abdominal obesity, BMI, waist circumference, cardiovascular diseases, visceral fat

Introduction

Cardiovascular diseases (CVDs) are among the leading causes of death worldwide (1). In 2019, the World Health Organisation estimated annual cases of 17.9 million deaths due to CVDs, which accounted for 32% of all deaths globally (2). In Indonesia, CVDs are also among the top three causes of disability-adjusted life years (3). It is well established that obesity is a risk factor for CVDs, and with more than one-third (35%) of the global adult population having overweight or obesity (4), these individuals are also becoming more susceptible to developing the conditions.

The development of CVDs from obesity involves several simultaneous pathways. A common explanation is elevated cardiac output and eventually blood pressure (hypertension) due to increased demand for oxygen and nutrients from body organs in overall obesity (5). Chronic hypertension changes the anatomical structure and physiological functions of the heart due to maladaptive remodeling, causing cardiac enlargement and muscle thickening to maintain the ejection fraction, which are causes of CVDs (6). It is also known that the negative health consequences of obesity are not only influenced by the excess fat mass, but also by the sites in the body where the fat is accumulated (7). In particular, fat deposition in the abdominal region, known as visceral fat, is associated with detrimental cardiometabolic health outcomes, a pathway that may explain the development of CVDs from abdominal obesity (8). Visceral fat accumulation in the abdomen leads to liver exposure to high concentrations of free fatty acids and glycerol, which in turn increases hepatic production of triglyceride-rich circulating lipoproteins (9). This promotes atherosclerosis, or cholesterol plaque buildup in the vascular system, which may result in myocardial infarction or stroke (10). However, although these possible pathways from overall and abdominal obesity are known, the relative contributions from the two phenotypes of obesity were not often measured quantitatively and comparatively.

To date, large epidemiological studies exploring the associations between body fat distribution and the risk of CVDs in the Asian population were still rare, despite the well-known fact that the Asian populations have higher cardiometabolic risks within the same amount of adiposity compared with the Westerns (11,12), urging etiological research and public health measures for this population. To what extent overall and abdominal obesity

coexist in Asian individuals was also not much known, and whether the separate or joint presence of them is differentially associated with the cardiovascular risks in men and women also remains unclear, despite the differences in body fat distribution between sexes (13,14), which are generally described by the so-called 'apple-shaped' or 'pear-shaped' adiposity (15).

Therefore, in the present study, we aimed (1) to estimate the 10-year CVD risks in the Indonesian population, and (2) to investigate how the separate and joint presence of overall and abdominal obesity were associated with the 10-year risk. Using the large, nationally representative data from the Indonesian National Health Survey, we analysed the population as stratified by sex, age, and urban/rural characteristics to gain insight into how these characteristics play a part in the associations between overall or abdominal obesity and CVD risks.

Methods

This study is a cross-sectional analysis of the Indonesian National Health Survey, which is a nationwide survey that was conducted by the Ministry of Health, Government of Indonesia. The population-based survey aimed to monitor the general health status of the citizens and screen for the presence of infectious, cardiometabolic, and degenerative diseases. The survey is held periodically every five years, with the 2018 survey being the most recent one with publicly available data, which we analysed in this study. To ensure the sample representativeness of the general Indonesian population, the national survey applied a multi-stage sampling method combining cluster and systematic random sampling, as well as probability proportional to size methods to select households in the 34 provinces. The national survey also applied weighting factors to account for differences in the populational density and urban-rural distribution across the provinces; hence, the results apply to the whole Indonesian population with its diverse sociodemographic characteristics.

Households were the smallest sampling unit in the national survey, and all individuals or family members were included as participants if their households were selected. Data collection was performed during a household visit, in which healthcare personnel from local Community Health Centres asked participants to complete an interviewer-assisted questionnaire to obtain information on their sociodemographic characteristics, lifestyle, and

general health status, including medical history of CVDs. In a weighted and randomly selected subsample of participants, a blood sample was also taken for a glucose and lipid laboratory test. The national survey design and population are described elsewhere in detail (16,17). The Health Research Ethics Committee of NIHRD, Ministry of Health, Republic of Indonesia, approved the design of the national survey (LB.02.01/2/KE.267/2017). All participants signed written informed consent, and all data were analysed anonymously. The ethics of this present study have been reviewed and approved by The Research and Community Engagement Ethical Committee, Faculty of Public Health, Universitas Indonesia (No: Ket-152/UN2.F10.D11/PPM.00.02/2024).

From 1,017,290 individuals who were sampled as participants in the national health survey, in this study we only included non-pregnant individuals aged ≥15 years who were randomly selected for blood laboratory tests (n=33,786). We further excluded those aged <30 years and those with a prior history of CVDs (n=6,738), as well as those with incomplete biomarkers data (n=433), resulting in a total number of 26,615 participants. The selection of participants in the present study is illustrated in **Supplemental Figure 1.**

The 10-year CVD Risk: The Framingham Risk Score

We calculated the 10-year CVD risk with the Framingham score, as the percent-risk of developing the disease in ten years. The Framingham Risk Score is the most widely used model used by clinicians worldwide to predict the likelihood of CVD incidence in individuals without a prior history of the disease (18). The score used several clinical characteristics as predictors to estimate the 10-year CVD risk: age, blood pressure, HDL and total cholesterol, and smoking history. It distinguishes the different risks between men and women, giving the two sexes different coefficients for each predictor. The risks of developing CVD in ten years are further stratified into low-risk (<10%), moderate risk (10-20%), and high-risk (>20%). The detailed scoring of the Framingham Risk Score is available in its original publication (19).

Anthropometric Measurements of Obesity

To assess the body fat distribution, we used the estimates of overall and abdominal obesity as the study exposures. Overall obesity is measured with BMI, which is an individual's body

weight in kilograms divided by the square of his height in meters. The body weight was measured with a digital FESCO ™ weight scale without shoes to the nearest 0.1 kg, whereas the height was measured with a wall-fixed stadiometer to the nearest 0.1 cm. Abdominal obesity is assessed with waist circumference, which is measured halfway between the iliac crest and the lowest rib with a flexible tape to the nearest 0.1 cm.

Overall obesity was defined as a BMI of $\geq$25.0 kg/m^2, according to WHO Asia-Pacific criteria for obesity (20). Individuals with a BMI ranging from 23.0 to 24.9 kg/m^2 were considered overweight, and those with a BMI of <23.0 kg/m^2 were considered normal weight. Abdominal obesity was defined as waist circumference above ethnic-specific cut-offs, which are $\geq$90 cm for men and $\geq$80 cm for women in the Asian population (12,21).

Clinical Biomarkers

From the national survey population, blood venous plasma samples were taken in a randomly selected and weighted subsample of participants. The puncture was made in the left cubital fossa while the participants were seated. The blood sampling was performed in the morning after the participants were asked to fast overnight. Blood lipid profiles were analysed with the standard chemical chemistry method, while blood glucose levels were measured with a rapid Accu-Check ™ glucometer from a capillary sample. Blood pressure was measured in the left upper arm while the participants were seated after five minutes of rest. The average blood pressure from three measurements was calculated and used in the analyses (16,17).

Statistical Analyses

Descriptive characteristics were presented as mean (SD) or median (25th–75th percentiles) for continuous variables, and as proportion (%) for categorical variables. As all analyses were weighted to account for the sampling design, the number of participants in the stratified groups could not be obtained, but a percentage was given instead.

To investigate the separate associations of overall or abdominal obesity with the 10-year CVD risks, we performed multivariable linear regressions using the standardised unit of BMI and waist circumference (WC) as the exposures. Effect sizes were presented as regression coefficients (β) with 95% confidence intervals, and the associations were adjusted for

sociodemographic confounding factors (age, sex, education, occupation, marital status, and urban/rural living situation).

To investigate which one had a stronger relative contribution to CVD risks, we examined the joint associations of overall obesity (OO) versus abdominal obesity (AO) to the 10-year risks. The association between BMI-CVD risks was mutually adjusted for WC, while the WC-CVD relationship was adjusted for BMI. In addition, we created four combined exposure groups: OO-/AO-(reference), OO+/AO-, OO-/AO+, and OO+/AO+, and multivariable linear regressions were performed to calculate the β (95% CIs) for each group compared to the reference category, adjusted for confounding factors.

To examine whether the associations may differ between sexes, age groups, and urban/rural population settings, we repeated the multivariable linear regression analysis in these subgroups separately. These stratified analyses were done to gain insight into the role of sex and urban/rural differences in the associations between body fat distribution and CVD risks, as previous studies have shown that visceral fat was more strongly associated with insulin resistance and subclinical atherosclerosis in women than men (22,23), and that urban living situations were associated with higher risks of developing CVD than rural settings (24,25).

Results

Descriptive Characteristics of the Study Population

The sex distribution was similar in the study population (Men: 50.6%; Women: 49.4%), and approximately half of the individuals (54.2%) lived in urban settings. The mean (SD) age of participants was 49.3 (12.3) years. Approximately six-percent participants were highly educated, and they lived more often in urban than rural areas [Supplemental Table 1a]. Half of women (51.7%) were unemployed or stay-at-home mothers, whereas the unemployment rate was 9.2% for men. Two-thirds of men (66.2%) were smokers, whereas women rarely smoke. Hypertension was common in the population (%), and the mean (SD) of systolic [135.8 (24.7) mmHg] and diastolic [85.9 (13.2)] blood pressure were above normal cut-offs. In regard to the prevalence of obesity, men had only half the prevalence of overall obesity

[Men: 26.3%; Women: 50.1%], and one-third of the prevalence of abdominal obesity [Men: 20.5%; Women: 58.7%], than women [Table 1].

The overall and abdominal obesity rates in urban areas were approximately 1.5 times higher than in rural areas. Compared to individuals without obesity, those with (abdominal) obesity have higher average blood pressure levels, total cholesterol, and triglycerides. Other characteristics of the population as stratified by urban/rural living situations and obesity phenotypes were described in Supplemental Tables 1a and 1b.

The Estimated 10-Year CVD Risks

When stratifying the population by sex, men [14.3 (8.9) %] have almost double the risk of developing CVDs in ten years than women [8.0 (9.3) %] of all ages. Among these, 37.3 % of men and 14.1 % of women have high risks (>20%) of CVDs. When stratifying the population according to urban or rural living situation, the mean (SD) of 10-year CVD risks were not different [Urban: 11.1 (9.4); Rural: 11.3 (10.1) %] in individuals living in urban and rural areas, with similar proportions of those in the high-risk category [Urban: 25.7%; Rural: 25.2%]. In regard to age, more than two-thirds of individuals aged sixty years and older (60–69, 70–70, $\geq$80) had high risks (>20%) of developing CVDs in ten years. The complete subgroup estimations of the 10-year CVD risks according to sex-, urban/rural-, obesity phenotypes, and age stratifications were presented in Table 2.

The associations of overall and abdominal obesity with the 10-year CVD risks

In both men and women, abdominal adiposity [adjusted β (95% CI): 0.95 (0.72-1.18) in men and 1.06 (0.87-1.24) in women] was more strongly associated with the 10-year CVD risks than overall adiposity [0.75 (0.50-1.01) in men; 0.43 (0.25-0.61) in women] [Table 3]. Additional adjustment for waist circumference strongly attenuated the association between BMI and the 10-year CVD risks, but not so much when the model with waist circumference was mutually adjusted for BMI, implying the higher relative contributions of abdominal obesity to the 10-year CVD risks [Table 3].

After stratifying by urban/rural living situation, this pattern of associations was also observed in both urban and rural populations [Supplemental Table 2a]. After stratifying by age groups,

the strong association between abdominal adiposity and the 10-year CVD risk was also observed in the middle-aged (40–70 years), but not so much in the younger (aged<40) or older (aged>70) adults [Supplemental Table 2b].

The Joint Effect of Overall and Abdominal Obesity on the 10-Year CVD Risks

Compared to individuals without any obesity (neither overall nor abdominal obesity) who were set as the reference group, those with both overall and abdominal obesity coexisted had a three-percent higher risk of developing CVDs in ten years [adjusted β (95% CI): 3.00 (2.80-3.21)]. Overall and abdominal obesity coexisted in 30.7% of individuals in the population, whereas the proportions of those who had only overall or abdominal obesity were 7.4% and 8.7%, respectively. Those with only abdominal obesity had a 1.82 (1.50–2.15) higher percent-risk, whereas those with only overall obesity had a 1.44 (1.14–1.74) higher percent-risk, of developing CVDs in ten years compared to those without obesity [Table 4].

Discussion

In this nationally representative study which analyzed large-scale data from the Indonesian National Health survey, we estimated the 10-year CVD risks in the Indonesian population. We also examined the associations of overall and abdominal obesity to the 10-year disease risks, and investigated which obesity phenotype has a stronger association with the cardiovascular risks. We observed that men had almost double the risk than women of developing CVDs in ten years. Men also had more tendencies to be in the high-risk (>20% risks) category than women. On the contrary, overall and abdominal obesity were both more prevalent in women than in men. Both overall and abdominal obesity were associated with the 10-year CVD risks, but the association between overall obesity and the cardiovascular disease risks strongly attenuated after mutual adjustment for abdominal obesity, implying the higher relative contribution of abdominal adiposity in explaining the 10-year CVD risks.

The higher risk of CVD in men, despite the lower prevalence of obesity, may be explained by the fact that men had a smoking habit that was disproportionately higher than women. It is well known from the literature that smoking raises triglycerides and lowers HDL, which is a predisposing factor for atherosclerosis (26,27). Previous studies from other populations

worldwide also supported the higher burden of CVDs in men than that of women. Men were more affected by premature death due to CVDs, as the risks were higher than women of the same age (28,29).

Nevertheless, when we look at how obesity impacts CVD risks, this study shows that women suffer more from the detrimental cardiovascular effects of obesity, as they have stronger associations between obesity and CVD risks. This is more particularly so for abdominal than overall obesity, because the association between overall obesity and CVD is strongly attenuated after adjustment for abdominal obesity, which implies that fat accumulation in the abdomen strongly explains the association between overall adiposity and CVD. This sex discrepancy in cardiovascular risks within the same amount of (abdominal) adiposity was also observed in previous studies, which shows that visceral fat was more strongly associated with insulin resistance and subclinical atherosclerosis in women than men (22,23).

In the present study, we did not observe a pronounced difference in the 10-year CVD risks between urban versus rural populations, despite the higher prevalence of obesity in urban areas. This may be explained by the fact that, despite urbanization having been strongly linked to increased cardiometabolic risks due to physical inactivity and ultra-processed food consumption, the risks were also rising in the rural population due to older demographics and socioeconomic disadvantages (30,31). Obesity was also not differentially related to CVD in urban and rural populations, probably because the sex distribution was similar in the two settings.

The strength of this study is the large sample size from the Indonesian National Health Survey, which enabled us to perform subgroup analyses and supports the generalization of study results to the whole Indonesian population (16,17). However, there are several limitations of the present study. First, due to the observational nature of this study, this implied that residual confounding was still possible. However, to lessen this shortcoming, we had adjusted every known and measured confounding factor in the multivariable regression models. Second, although the national survey is held periodically every five years, each survey was cross-sectional by design, as the sample population was randomly selected in each survey. Therefore, no longitudinal time-to-event data was available, and the actual incidence of CVD cannot be examined. Hence, we could only use the estimated 10-year risk

from a prediction model. Due to the cross-sectional design, we could not also suggest any causality. However, reverse causation is unlikely as the causal association of obesity to CVD is well established from previous literature (32,33). Nevertheless, despite the mentioned limitations, this study is still the most representative of the general Indonesian population, as extensive cohort studies in Indonesia are still rare, particularly with a population as large as this survey.

This study has several public health and research implications. First, the stronger relative contribution of abdominal obesity over overall obesity to the CVD risks implies the importance of highlighting abdominal obesity in preventing CVDs. This poses a challenge as an earlier study has shown that the general public is less aware of the meaning of abdominal obesity and the negative health consequences that pertain to it (34). Hence, raising public awareness on the definition, measurement, and prevention of abdominal obesity to reduce the risk of CVD in the population is crucial, and routine measurement of waist circumference in daily clinical practice may become a practical but fundamental starting point. Nevertheless, a previous study revealed that waist circumference measurement was not performed routinely by general practitioners in their daily clinical practices, and thus, reinforcing its routine measurements is necessary (35). Second, as this study revealed the more detrimental cardiovascular effect of (abdominal) obesity in women than in men, attention or targeted intervention to prevent obesity and its impact on women should be prioritized, especially because earlier studies have shown the higher prevalence of obesity in adult women than men, particularly among the Asian population (36,37). Sex differences should also be taken into account when designing public health policies related to obesity and CVDs. Lastly, as in this study, we could only measure abdominal obesity as a proxy for visceral adiposity; we also recommend further (longitudinal) studies with direct measure of visceral fat (e.g., with MRI or CT scan) to straightly investigate how visceral adiposity impacts CVD risks differentially in men and women.

Taken together, to conclude this nationally representative study, we observed that although men had almost double the risk than women of developing CVD in ten years, women had a higher prevalence of obesity and suffered more from the detrimental cardiovascular effects of obesity than men. Abdominal obesity, presumably explained by visceral fat accumulation

in the abdomen, was more strongly associated with an increased risk of CVD than overall adiposity, and more particularly so in women than men. Raising awareness of the importance of abdominal obesity in clinical practice may benefit in preventing CVD and its related morbidity and mortality. Further studies and public health policies to address the more damaging cardiovascular effects of abdominal/visceral adiposity in women than in men are necessary.

Authors Statement

The authors declare no conflict of interest. The partial results of this study was presented orally at the 2023 ICOMES Symposium and won the '*Excellent Oral Presentation Award*'. The complete analysis results within this full, written manuscript have never been published anywhere. The publication of this paper is funded by the Directorate of Research and Development, Universitas Indonesia, under Hibah PUTI 2024.

References

1. World Health Organization. The top 10 causes of death [Internet]. [cited 2021 Oct 18]. Available from: https://www.who.int/news-room/fact-sheets/detail/the-top-10-causes-of-death

2. World Health Organization. Cardiovascular diseases (CVDs) [Internet]. [cited 2021 Oct 18]. Available from: https://www.who.int/news-room/fact-sheets/detail/cardiovascular-diseases-(cvds)

3. Mboi N, Murty Surbakti I, Trihandini I, Elyazar I, Houston Smith K, Bahjuri Ali P, et al. On the road to universal health care in Indonesia, 1990-2016: a systematic analysis for the Global Burden of Disease Study 2016. Lancet. 2018 Aug 18;392(10147):581–91.

4. World Health Organization. Obesity and overweight [Internet]. [cited 2021 Sep 9]. Available from: https://www.who.int/news-room/fact-sheets/detail/obesity-and-overweight

5. Ashraf MJ, Baweja P. Obesity: the 'huge' problem in cardiovascular diseases. Mo Med. 2013 Dec;110(6):499–504.

6. Saheera S, Krishnamurthy P. Cardiovascular Changes Associated with Hypertensive Heart Disease and Aging. Cell Transplant. 2020 Dec;29:963689720920830.

7. Després JP, Lemieux I. Abdominal obesity and metabolic syndrome. Nature. 2006 Dec 14;444(7121):881–7.

8. Tchernof A, Després JP. Pathophysiology of human visceral obesity: an update. Physiol Rev. 2013 Jan;93(1):359–404.

9. Neeland IJ, Ross R, Després JP, Matsuzawa Y, Yamashita S, Shai I, et al. Visceral and ectopic fat, atherosclerosis, and cardiometabolic disease: a position statement. Lancet Diabetes Endocrinol. 2019 Sep;7(9):715–25.

10. Dutta P, Courties G, Wei Y, Leuschner F, Gorbatov R, Robbins CS, et al. Myocardial infarction accelerates atherosclerosis. Nature. 2012 Jul 1;487(7407):325–9.

11. Sniderman AD, Bhopal R, Prabhakaran D, Sarrafzadegan N, Tchernof A. Why might South Asians be so susceptible to central obesity and its atherogenic consequences? The adipose tissue overflow hypothesis. International Journal of Epidemiology. 2007 Feb 1;36(1):220–5.

12. Alberti KGMM, Eckel RH, Grundy SM, Zimmet PZ, Cleeman JI, Donato KA, et al. Harmonizing the metabolic syndrome: a joint interim statement of the International Diabetes Federation Task Force on Epidemiology and Prevention; National Heart, Lung, and Blood Institute; American Heart Association; World Heart Federation; International Atherosclerosis Society; and International Association for the Study of Obesity. Circulation. 2009 Oct 20;120(16):1640–5.

13. Kanter R, Caballero B. Global Gender Disparities in Obesity: A Review. Advances in Nutrition. 2012 Jul 1;3(4):491–8.

14. Zore T, Palafox M, Reue K. Sex differences in obesity, lipid metabolism, and inflammation-A role for the sex chromosomes? Mol Metab. 2018 Sep;15:35–44.

15. Fu J, Hofker M, Wijmenga C. Apple or Pear: Size and Shape Matter. Cell Metabolism. 2015 Apr 7;21(4):507–8.

16. National Institute for Health Research and Development (NIHRD), Ministry of Health, Republic of Indonesia. Laporan Nasional RISKESDAS 2018 [Internet]. 2018. Available from: https://repository.badankebijakan.kemkes.go.id/id/eprint/3514/

17. National Institute for Health Research and Development (NIHRD), Ministry of Health, Republic of Indonesia. RISKESDAS Tahun 2018 [Internet]. [cited 2021 Oct 18]. Available from: https://layanandata.kemkes.go.id/katalog-data/riskesdas/ketersediaan-data/riskesdas-2018

18. Mohd Faizal AS, Thevarajah TM, Khor SM, Chang SW. A review of risk prediction models in cardiovascular disease: conventional approach vs. artificial intelligent approach. Computer Methods and Programs in Biomedicine. 2021 Aug 1;207:106190.

19. D'Agostino RBS, Vasan RS, Pencina MJ, Wolf PA, Cobain M, Massaro JM, et al. General cardiovascular risk profile for use in primary care: the Framingham Heart Study. Circulation. 2008 Feb 12;117(6):743–53.

20. World Health Organization, Western Pacific Region. The asia-pacific perspective: redefining obesity and its treatment. Health Communications Australia; 2000.

21. Internatonal Diabetes Federation. IDF Consensus Worldwide Definition of the Metabolic Syndrome [Internet]. [cited 2021 Sep 10]. Available from: https://idf.org/about-diabetes/resources/

22. de Mutsert R, Gast K, Widya R, de Koning E, Jazet I, Lamb H, et al. Associations of Abdominal Subcutaneous and Visceral Fat with Insulin Resistance and Secretion Differ Between Men and Women: The Netherlands Epidemiology of Obesity Study. Metab Syndr Relat Disord. 2018 Feb;16(1):54–63.

23. Gast KB, den Heijer M, Smit JWA, Widya RL, Lamb HJ, de Roos A, et al. Individual contributions of visceral fat and total body fat to subclinical atherosclerosis: The NEO study. Atherosclerosis. 2015 Aug;241(2):547–54.

24. Han B, Chen Y, Cheng J, Li Q, Zhu C, Chen Y, et al. Comparison of the Prevalence of Metabolic Disease Between Two Types of Urbanization in China. Front Endocrinol (Lausanne). 2018 Nov 12;9:665–665.

25. Wang Y, Tu R, Yuan H, Shen L, Hou J, Liu X, et al. Associations of unhealthy lifestyles with metabolic syndrome in Chinese rural aged females. Sci Rep. 2020 Feb 17;10(1):2718.

26. Nakamura M, Yamamoto Y, Imaoka W, Kuroshima T, Toragai R, Ito Y, et al. Relationships between Smoking Status, Cardiovascular Risk Factors, and Lipoproteins in a Large Japanese Population. J Atheroscler Thromb. 2021 Sep 1;28(9):942–53.

27. van der Plas A, Antunes M, Pouly S, de La Bourdonnaye G, Hankins M, Heremans A. Meta-analysis of the effects of smoking and smoking cessation on triglyceride levels. Toxicology Reports. 2023 Jan 1;10:367–75.

28. Zhang J, Jin Y, Jia P, Li N, Zheng ZJ. Global Gender Disparities in Premature Death from Cardiovascular Disease, and Their Associations with Country Capacity for Noncommunicable Disease Prevention and Control. Int J Environ Res Public Health. 2021 Oct 2;18(19).

29. Kundu J, James KS, Hossain B, Chakraborty R. Gender differences in premature mortality for cardiovascular disease in India, 2017–18. BMC Public Health. 2023 Mar 23;23(1):547.

30. Schopfer DW. Rural health disparities in chronic heart disease. Preventive Medicine. 2021 Nov 1;152:106782.

31. Lindroth M, Lundqvist R, Lilja M, Eliasson M. Cardiovascular risk factors differ between rural and urban Sweden: the 2009 Northern Sweden MONICA cohort. BMC Public Health. 2014 Aug 9;14:825.

32. Limpijankit T, Vathesatogkit P, Matchariyakul D, Wiriyatanakorn S, Siriyotha S, Thakkinstian A, et al. Causal relationship of excess body weight on cardiovascular events through risk factors. Scientific Reports. 2022 Mar 28;12(1):5269.

33. Larsson SC. Causal association between adiposity and cardiovascular disease. European Heart Journal. 2019 Sep 14;40(35):2937–8.

34. Sigit FS, de Mutsert R, Lamb HJ, Meuleman Y, Kaptein AA. Illness perceptions and health-related quality of life in individuals with overweight and obesity. Int J Obes (Lond). 2022 Feb;46(2):417–26.

35. Dunkley AJ, Stone MA, Patel N, Davies MJ, Khunti K. Waist circumference measurement: knowledge, attitudes and barriers in patients and practitioners in a multi-ethnic population. Family Practice. 2009 Oct 1;26(5):365–71.

36. Prasad DS, Kabir Z, Revathi Devi K, Peter PS, Das BC. Gender differences in central obesity: Implications for cardiometabolic health in South Asians. Indian Heart J. 2020 Jun;72(3):202–4.

37. Awasthi A, Panduranga AB, Deshpande A. Prevalence of overweight/obesity in South Asia: A narrative review. Clinical Epidemiology and Global Health. 2023 Jul 1;22:101316.

Table 1. Descriptive Characteristics of the Indonesian Population, Sex-Stratified (n=26,615)

	**Total (100%)**	**Men (50.6%)**	**Women (49.4%)**
Age, *years*	49.3 (12.3)	50.2 (11.3)	48.3 (13.0)
Population Setting, *%Urban*	54.2	53.7	54.7
Education, *%High*	5.7	6.5	5.0
Occupation, *%Unemployed*	30.2	9.2	51.7
Marital Status, *%Married*	85.4	90.1	80.5
Smoking, *%yes*	35.2	66.2	3.3
Systolic Blood Pressure, *mmHg*	135.8 (24.7)	134.2 (21.0)	137.4 (28.4)
Diastolic Blood Pressure, *mmHg*	85.9 (13.2)	84.5 (11.7)	87.3 (14.5)
HDL Cholesterol, *mg/dL*	47.6 (11.4)	44.3 (9.3)	51.0 (12.5)
LDL Cholesterol, *mg/dL*	125.9 (33.4)	122.5 (29.1)	129.3 (37.4)
Total Cholesterol, *mg/dL*	186.4 (39.1)	180.8 (34.3)	192.1 (43.0)
Triglyceride, *mg/dL*	137.5 (100.0)	149.0 (102.5)	125.6 (90.6)
BMI, kg/m^2	24.1 (4.7)	22.9 (3.8)	25.4 (5.3)
Overall Obesity, *%yes*	38.1	26.3	50.1
Waist Circumference, *cm*	81.4 (12.1)	80.4 (10.8)	82.4 (13.3)
Abdominal Obesity, *%yes*	39.4	20.5	58.7

Data were presented as mean (SD) for continuous variables, or proportion (%) for categorical variables.

Table 2. The Stratified 10-Year Cardiovascular Disease Risks of the Indonesian Population, based on the Framingham Risk Score (n=26,615)

		Sex		Living Situation		Obesity Phenotypes				Age Group (Years)					
	**Total (100%)**	**Men (50.6%)**	**Women (49.4%)**	**Urban**	**Rural**	**OO-, AO-**	**OO+, AO-**	**OO-, AO+**	**OO+, AO+**	**30-39**	**40-49**	**50-59**	**60-69**	**70-79**	**≥80**
Mean (SD) 10-Year CVD Risks	11.2 (9.7)	14.3 (8.9)	8.0 (9.3)	11.1 (9.4)	11.3 (10.1)	11.6 (9.5)	10.5 (8.9)	11.4 (10.8)	10.6 (9.9)	2.9 (2.7)	7.7 (6.0)	15.0 (8.8)	20.4 (8.8)	24.5 (7.4)	24.9 (7.4)
Proportion (%) with															
Low Risk	69.6	55.7	83.1	70.7	68.4	65.4	77.4	70.5	75.6	99.6	91.2	50.0	18.8	6.7	5.8
Moderate Risk	4.9	7.0	2.8	3.6	6.4	8.6	-	1.6	-	0.1	2.1	9.1	12.3	9.8	9.7
High Risk	25.5	37.3	14.1	25.7	25.2	26.0	22.6	27.9	24.4	0.3	6.7	40.9	68.9	83.5	84.5

Data are presented as mean (SD) for continuous variables, or proportion (%) for categorical variables. The 10-year Cardiovascular Diseases risks were calculated using the Framingham Risk Score. The score was calculated as percent-risk (%), in which individuals with <10% were considered low risk, 10-19% as moderate risk, and >20% as having a high risk of developing CVD in ten years.

Table 3. The Associations of Overall and Abdominal Obesity with the Sex-stratified 10-Year Cardiovascular Diseases Risks (n=26,615)

		Total Population	Men	Women
**Overall Obesity**	Crude β (95% CI)	-0.70 (-0.84,-0.57)	0.11 (-0.12,0.34)	0.17 (0.02-0.31)
BMI	Adjusted β (95% CI)	1.40 (1.29-1.50)	1.53 (1.35-1.72)	1.24 (1.12-1.35)
SD = 4.7 kg/m^2	+Waist Circumference	0.67 (0.52-0.82)	0.75 (0.50-1.01)	0.43 (0.25-0.61)
		**Total Population**	**Men**	**Women**
**Abdominal Obesity**	Crude β (95% CI)	0.74 (0.60-0.88)	0.93 (0.72-1.15)	1.11 (0.95-1.26)
Waist Circumference	Adjusted β (95% CI)	1.41 (1.32-1.50)	1.46 (1.32-1.60)	1.39 (1.27-1.51)
SD = 12.1 cm	+BMI	0.92 (0.77-1.07)	0.95 (0.72-1.18)	1.06 (0.87-1.24)

Data were presented as regression coefficient (β) with 95% confidence interval (CI). Multivariate models were adjusted for age, sex, education, occupation, marital status, and living situation (urban/rural). The associations were additionally mutually adjusted for waist circumference and BMI.

Table 4. The Joint Effect of Overall and Abdominal Obesity on the 10-Year Cardiovascular Diseases Risks (n=26,615)

		OO-, AO- (53.2%)	OO+, AO- (7.4%)	OO-, AO+ (8.7%)	OO+, AO+ (30.7)
**Total Population**	Crude	1 (Reference)	-1.14 (-1.62, -0.66)	-0.25 (-0.72, 0.22)	-1.04 (-1.34,-0.74)
	Adjusted		1.44 (1.14-1.74)	1.82 (1.50-2.15)	3.00 (2.80-3.21)
		**OO-, AO- (70.3%)**	**OO+, AO- (9.2%)**	**OO-, AO+ (3.4%)**	**OO+, AO+ (17.1)**
**Men**	Crude	1 (Reference)	-0.89 (-1.53,-0.25)	4.05 (2.97-5.13)	2.05 (1.50-2.60)
	Adjusted		1.46 (1.07-1.85)	1.73 (0.99-2.47)	3.20 (2.85-3.55)
		**OO-, AO- (35.7%)**	**OO+, AO- (5.6%)**	**OO-, AO+ (14.2%)**	**OO+, AO+ (44.5)**
**Women**	Crude	1 (Reference)	-0.80 (-1.38,-0.21)	2.78 (2.28-3.28)	1.46 (1.15-1.78)
	Adjusted		1.48 (1.06-1.91)	1.96 (1.59-2.33)	2.78 (2.54-3.02)

OO, Overall Obesity; AO, Abdominal Obesity. Data were presented as regression coefficient (β) with 95% confidence interval (CI). Multivariate models were adjusted for age, sex, education, occupation, marital status, and living situation (urban/rural).

Supplementary Tables and Figures

Click here to access/download
Supporting Information
Supplementary Tables & Figures.docx
